# Grand Challenges of Translational Research in Rehabilitation

**DOI:** 10.3389/fresc.2021.625055

**Published:** 2021-03-24

**Authors:** Chetwyn C. H. Chan

**Affiliations:** ^1^Department of Rehabilitation Sciences, The Hong Kong Polytechnic University, Hong Kong, China; ^2^University Research Facility in Behavioral and Systems Neuroscience, The Hong Kong Polytechnic University, Hong Kong, China

**Keywords:** translational research, rehabilitation science, basic science, constraint-induced movement therapy, multisensory integration, Ayres sensory integration

## Translational Research in the Context of Rehabilitation

Translational research can simply be understood as “bench to bedside,” or translating new knowledge into application. Fort et al. (1) classified translational research into five different stages, spanning from basic biomedical research (T0) to outcomes and effectiveness in population (T4). Herein, we define translational rehabilitation research as making use of findings from basic or theoretical research to inform assessments and interventions that benefit individuals with disabilities. In the context of the *Frontiers in Rehabilitation Sciences* eight-theme framework, we focus on three specific stages, T0, T1, and T2: creating theoretical knowledge (T0), testing ideas in people with or without disabilities (T1), and establishing effectiveness and clinical guidelines (T2). The purpose of this paper is to review common issues in the design and delivery of two therapeutic approaches to client groups to illustrate the significant challenges of translational research in rehabilitation. These are the constraint-induced movement therapy services for stroke survivors and sensory integration for children with autism spectrum disorders (ASDs). The reasons for using these two approaches as examples are: first, their mechanisms tap on theories related to neuroplasticity and learning, and second, they have been commonly adopted for use by clinicians and can be easily accessed by clients around the world.

## Cimt for Stroke Survivors

Post-stroke rehabilitation plays an important role in facilitating functional regain for the participation and activity of stroke survivors. In the past two decades, the clinical intervention that has received the most attention has been constraint-induced movement therapy (CIMT). The original idea of CIMT was to tackle the learned non-use phenomenon among post-stroke patients (2). Since then, more than 50 randomized controlled trials have been published reporting the effectiveness of CIMT [e.g., (3, 4)]. Conducting randomized controlled trials is part of demonstrating treatment efficacy, which is the T2 stage of translational research. According to Fort et al. (1), T2 stage is in the middle of the translation process which is a stage subsequent to T0 (creating theoretical knowledge) and T1 stages (testing ideas in people). In this case, CMIT may be regarded as an effective clinical intervention for serving post-stroke survivors but not grounded from well-developed theoretical basis.

In a meta-analytic review, Kwakkel et al. (5) concluded that CIMT showed strong evidence supporting its effectiveness for improving the upper limb paresis of patients due to stroke. These functions include motor function, arm-hand activities, and self-reported arm-hand functioning in daily life, of which the enhancement effects referred to motor learning principles or repetitive training. However, in the same paper, Kwakkel et al. also concluded that no evidence was found on the type of CIMT, dosage, or timing influencing the treatment outcome. More importantly, the authors pointed out that the mechanisms underlying CIMT effect remain unclear. For instance, they suggested future study to investigate the mechanism underlying “learned misuse” which is one main assumption of CIMT. Along a similar line, an earlier study conducted by Gauthier et al. (6) offered an alternative explanation for the CIMT treatment effect. By comparing CIMT with a transfer package versus CIMT alone, the results indicated that the post-treatment gray matter changes in the participating patients were attributed to transfer package rather than CIMT. The transfer package involved participants to apply the gained limb functions to real world situations. Xu et al. (7) employed electromyogram and transcranial magnetic stimulation and reported interhemispheric imbalance, another main assumption of CIMT, failed to account for the post-stroke motor recovery among patients who received the intervention. Jiang et al. (8) revealed that CIMT was found to be relatively less effective and have larger variability in the effects in patients in the acute or subacute phase than in those in the chronic phase. The researchers explained that these observations were perhaps attributable to the varied brain activation patterns of patients, as well as their within-group clinical heterogeneity. The example of CIMT presented here illustrates a common issue in post-stroke rehabilitation that interventions available to clients are likely to be based on the evidence of the T2 stage but not of the T1 or T0 stage.

To further strengthen translational research on CIMT or other interventions, more questions related to the T0 and T1 stages will need to be asked. For instance, what are the brain recovery models after stroke, and how can these models be modified by individualized factors such as lesion-induced deficits and brain activation patterns (T0 stage)? What are the interactive roles of growth promoting and inhibiting factors influencing the recovery models (T0 stage)? Another important question is how the findings generated from animal studies at the molecular, cellular, and system levels can be generalized to human and patients (T1 stage). Researchers in different disciplines have begun addressing questions along these lines. Exploration on the effect of enriched environment promoting neurogenesis (9) and synaptic plasticity (10) using animal models contributes to theoretical knowledge corresponding to T0 stage work, while the effects of individual variability on recovery from brain injuries [e.g., (11)] corresponding to T1 stage work.

## Asi for Children

Besides stroke survivors, another group of individuals in high need of rehabilitation services are children with ASD who present with a wide range of learning, communication, and social disabilities [e.g., (12)]. Sensory integration (SI) has been identified as one of the core interventions for children with ASD (13). In the past 30 years or so, researchers and practitioners conducted studies on the therapeutic effects of SI. The results have been described as largely inconclusive [e.g., (14)]. Schoen et al. (13) conducted a comprehensive review and argued that one reason for the inconclusive findings was that the contents of the majority of SI interventions did not adhere to the principles of Ayres Sensory Integration (ASI). ASI stipulates clear principles, such as individualized tailoring, active engagement of the child, and establishing a therapeutic alliance between the child and therapist, as well as the ASI Fidelity Measure. In the same review, Schoen et al. identified 19 papers that passed the preliminary content and quality thresholds. However, among them, only three studies were selected to enter into the final review against the criteria set out in the Council for Exceptional Children (CEC) Standards for Evidence-based Practices in Special Education. The CEC standards stipulate the quality indicators to evaluate whether the research design and methods for supporting the evidence-based practice of an intervention are satisfactory. The findings of the review were that the three studies met 100, 85, and more than 50% of the CEC standard items, respectively.

ASI researchers have examined the T2 stage in translational research, disseminating ASI principles and generating valid evidence on treatment efficacy. These noble efforts will take some time to consolidate to further strengthen evidence-based ASI practice. Researchers have also conducted studies building up the theoretical basis of ASI (i.e., the T0 and T1 stages). Noel et al. (12) revealed that individuals with ASD had an enlarged temporal binding window, which sheds light on their deficits in multisensory temporal acuity. Lane et al. (15) attempted to articulate the ASI principles with theories and research findings in neuroscience. The authors concluded that the ways ASI conceptualizes developmental functions and disabilities are consistent with parts of the theories and mechanisms in vestibular, proprioceptive, and tactile sensory systems. Kilroy et al. (16) associated sensory registration, modulation, and motivation within ASI with findings of current neuroimaging literature. The results indicate that emotion-related regions such as the amygdala and insula are associated with registration, increased activation in the amygdala associated with modulation, and reduced connectivity between primary sensory regions and the reward system associated with motivation. These studies pointed to the direction of using neuroimaging and neurophysiological methods to identify biomarkers, as well as adopting neuroplasticity to explain the treatment effects of ASI.

ASI researchers have taken a rather different approach to translational research when compared with those of CIMT. The CIMT have accumulated sufficient evidence on its clinical effectiveness (T2), while has plenty of room of establishing its theoretical basis (T0 and T1) (Scenario 1 in Table 1). The ASI attempts to link existing wealth of knowledge on neural development and plasticity (T0) to ASI principles (T1 stage) (Scenario 2 in Table 1). The ASI principles are for prescribing context and content of ASI intervention and clinical guidelines (T2). These initiatives somewhat re-position its translational process back to T0, T1, and then T2 stages, which in the long run will contribute to constructing ASI practice grounded from a solid theoretical basis. To further strengthen translational ASI research, researchers will need to ask more questions to strengthen the links among the T0, T1, and T2 stages. For instance, what is the role of genomics in ASD-related brain connectivity, and how do these abnormalities contribute to the developmental disabilities unique to this disorder (T0 and T1)? How plastic is the neural system of individuals with ASD in response to multisensory stimuli (T1 and T2)? What is the efficacy of ASI-based interventions for enhancing functional adaptability of individuals with ASD (T2)?

**Table 1:** Comparisons of common translational research statuses in rehabilitation.

	**Translational research stages**		
	**T0**	**T1**	**T2**
Scenario 1	+ −−−	+ −−−	+ + + −
Scenario 2	+ + + −	+ + −−	+ −−−

“*+” means with evidence support; “−” means without evidence support. T0, T1, and T2 are translational research stages described in Fort et al. (1); T0, creating theoretical knowledge; T1, testing ideas in people; and T2, establishing effectiveness and clinical guidelines*.

## Our Significant Challenges

The CIMT and ASI examples highlight some challenges in translational research in rehabilitation. The challenges faced by the researchers in stages T0 to T1 are different from those in stages T1 to T2. An earlier paper written by the Cumberland Consensus Working Group (17) described the mismatch of inspiration between scientists who focus on bench work and whose interests are mostly “curiosity-led” and clinicians who focus on tackling patients' problems and whose interests are mostly “effectiveness of the interventions.” The “translational research pipeline,” due to various mismatches, was stalled. In the past 10 years, we have witnessed strategies developed by various parties to tackle the pipeline issues. Major research grants emphasize translational research, such as the Medical Research Council of the United Kingdom and Translational Research Institute of Australia, for driving innovation and speeding up the transfer of the best ideas into new interventions. An increasing number of universities and research centers evaluate research achievements in terms of esteem measures, impact, and knowledge transfer. We should capitalize on these recent developments by inviting researchers and clinical scientists to share their works and ideas on moving basic science discoveries into human research in the *Translational Research in Rehabilitation* specialty.

The T1 to T2 stages involve dissemination of discoveries and findings on human subjects for uptake by clinicians and patients. Jett (18) proposed a few strategies of implementation research to bridge the gaps between the two groups of players. They include developing needs for change in clinical practice, designing interventions with evidence for change in practice, implementing interventions in collaboration with members of research teams and clinicians, and evaluating the outcomes of the interventions. Research papers and reports describing the processes and outcomes also form the main content of this specialty.

## Author Contributions

CC conceptualized and wrote the paper.

## Conflict of Interest

The author declares that the research was conducted in the absence of any commercial or financial relationships that could be construed as a potential conflict of interest.
